# Assessment of the protein interaction between coagulation factor XII and corn trypsin inhibitor by molecular docking and biochemical validation

**DOI:** 10.1111/jth.13773

**Published:** 2017-08-09

**Authors:** B. K. Hamad, M. Pathak, R. Manna, P. M. Fischer, J. Emsley, L. V. Dekker

**Affiliations:** ^1^ School of Pharmacy Centre for Biomolecular Sciences University of Nottingham Nottingham UK

**Keywords:** corn trypsin inhibitor, factor XII, molecular docking simulation, serine protease, trypsin

## Abstract

**Essentials**

- Corn Trypsin Inhibitor (CTI) is a selective inhibitor of coagulation Factor XII (FXII).
- Molecular modelling of the CTI‐FXIIa complex suggested a canonical inhibitor binding mode.
- Mutagenesis revealed the CTI inhibitory loop and helices α1 and α2 mediate the interaction.
- This confirms that CTI inhibits FXII in canonical fashion and validates the molecular model.

**Summary:**

**Background**

Corn trypsin inhibitor (CTI) has selectivity for the serine proteases coagulation factor XII and trypsin. CTI is in widespread use as a reagent that specifically inhibits the intrinsic pathway of blood coagulation but not the extrinsic pathway.

**Objectives**

To investigate the molecular basis of FXII inhibition by CTI.

**Methods**

We performed molecular docking of CTI, using its known crystal structure, with a model of the activated FXII (FXIIa) protease domain. The interaction model was verified by use of a panel of recombinant CTI variants tested for their ability to inhibit FXIIa enzymatic activity in a substrate cleavage assay.

**Results**

The docking predicted that: (i) the CTI central inhibitory loop P1 Arg34 side chain forms a salt bridge with the FXIIa S1 pocket Asp189 side chain; (ii) Trp22 from CTI helix α1 interacts with the FXIIa S3 pocket; and (iii) Arg43 from CTI helix α2 forms a salt bridge with FXIIa H1 pocket Asp60A. CTI amino acid substitution R34A negated all inhibitory activity, whereas the G32W, L35A, W22A and R42A/R43A substitutions reduced activity by large degrees of 108‐fold, 41‐fold, 158‐fold, and 100‐fold, respectively; the R27A, W37A, W39A and R42A substitutions had no effect. Synthetic peptides spanning CTI residues 20–44 had inhibitory activity that was three‐fold to 4000‐fold less than that of full‐length CTI.

**Conclusions**

The data confirm the validity of a canonical model of the FXIIa–CTI interaction, with helix α1 (Trp22), central inhibitory loop (Arg34) and helix α2 (Arg43) of CTI being required for effective binding by contacting the S1, S3 and H1 pockets of FXIIa, respectively.

## Introduction

Factor XII is a coagulation factor that circulates in plasma as an inactive zymogen 1. Cleavage of the Arg353–Val354 peptide bond generates the activated form FXIIa, which has a heavy chain of 50 kDa, connected to a light chain of 28 kDa by a Cys340–Cys467 disulfide bridge. The closest homolog of FXII is hepatocyte growth factor activator (HGFA), which has a very similar domain organization including a C‐terminal serine protease domain; a proline‐rich region is unique to FXII 2, 3, 4. FXIIa cleaves prekallikrein to generate kallikrein, which contributes to bradykinin generation 5. FXIIa also cleaves FXI to generate activated FXI (FXIa), which contributes to plasma coagulation 6. FXII knockout mice are protected against thrombosis and ischemic stroke in models of the disease, and it has been proposed that targeting FXII could result in medicines with a safer anticoagulation profile than the currently available anticoagulants; this has generated interest in developing selective inhibitors of FXIIa 7, 8.

Corn trypsin inhibitor (CTI) or corn Hageman factor inhibitor is a bifunctional serine protease and α‐amylase inhibitor 9, 10, 11, 12, 13, 14. It was first isolated from corn in the 1970s, and shown to have inhibitory activity against trypsin 12, 15, 16, 17, 18, 19. Subsequently, CTI was shown to potently inhibit FXIIa and moderately inhibit FXIa with selectivity over activated FX (FXa), thrombin, and kallikrein 10, 11, 14, 16, 17, 20. CTI is widely used to block the intrinsic pathway in *ex vivo* plasma studies of contact activation 17, 21. CTI could have utility as a coating agent to prevent contact activation in catheters 22.

The crystal structure of CTI reveals a central loop spanning residues 31–38 with an arginine at position 34 23. As this loop resembles a protease substrate site, it is proposed to act as an inhibition loop 23, 24. CTI also inhibits α‐amylase 25, through a site independent from the central inhibition loop predicted to interact with serine proteases 10, 11, and has antifungal activity 26, 27.

The preference of CTI for FXIIa and trysin is different from what has been determined for other inhibitors such as ecotin 28, which inhibits FXIIa, FXIa, trypsin, and thrombin. There are no known co‐crystal structures for CTI with trypsin or FXIIa to explain this. A non‐canonical binding mode has recently been proposed 29, implying that the inhibition loop of CTI is projected away from the active site in FXIIa. To understand CTI binding to and inhibition of FXIIa further, we used existing crystal structures for CTI and the FXII protease to generate a model for the complex, which we verified by mutagenesis of CTI, establishing a canonical model for CTI inhibition of FXIIa.

## Materials and methods

### Materials

Full length activated FXIIa (α‐FXIIa) and commercial CTI were obtained from Enzyme Research Laboratories (Swansea, UK). S2302 (a chromogenic substrate peptide mimic) was obtained from Chromogenix (Epsom, UK). A codon‐optimized CTI cDNA was obtained from GenScript (Piscataway, NJ, USA). High‐purity‐grade (> 95%) synthetic peptides were obtained from GenScript. Purity was confirmed by reverse‐phase HPLC and mass spectrometry. DNA primers were obtained from Eurofins MWG (Ebersberg, Germany).

### Docking of CTI and the FXII protease domain

The docking study was based on the available crystal structures of CTI 23, 24 (Protein Data Bank [PDB]: 1BFA and 1BEA) and on the crystal structure of the FXII protease in a zymogen‐like state that we previously described and termed FXIIac (PDB: 4XE4) and FXIIc (PDB: 4XDE). To generate a structure for the activated conformation of the FXIIa protease, a hybrid model of FXIIa was created with a similar approach to that used by previous authors 30. Step 1 used the crystal structure of closest homolog HGFA (PDB: 1YC0) as a template in the program swiss‐model 31, 32 to generate coordinates required for the active FXIIa S1 pocket (including residues 16–26, 133–147, 179–189, and 190–224; residue numbers according to the chymotrypsin numbering). In step 2, these coordinates were combined with the coordinates of the crystal structure of FXIIac (PDB: 4XE4) to generate the remainder of the FXIIa protease (Fig. S1). Unlike previous authors 30, who used the FXIIc coordinates (PDB: 4XDE), we utilized FXIIac (PDB: 4XE4), in which the closed H1 pocket is likely to be the dominant conformation 3. This model was regularized in coot, and superposed onto typical activated protease crystal structures of thrombin, trypsin, FXa and FXIa to check the positions of key conserved amino acids such as Asp189 in the S1 pocket and Trp215 in the S3 pocket, and to confirm that the N‐terminal Ile16 is correctly described. This was validated by inspection of a 4‐Å crystal structure of the activated β‐FXIIa protease (R. Manna and J. Emsley, unpublished data).

Docking was performed with this FXIIa hybrid model (referred to as the FXIIa protease) and CTI crystal structures (PDB: 1BFA and 1BEA, representing *Escherichia coli*‐expressed and native CTI protein crystal structures at resolutions of 2.2 Å and 1.95 Å, respectively), by use of the program cluspro with default parameters 33, 34, 35. To test the robustness of the docking algorithm, CTI docking calculations were performed with the related proteases trypsin, HGFA, thrombin, FXa, FXIa, activated FIX (FIXa), and activated FVII (FVIIa) 36. The top 10 docked complexes were inspected with pymol. When the pose was observed to be canonical (i.e. having the P1 CTI amino acid Arg34 docked into the protease S1 pocket), the relevant ranking position was recorded in Table S1. The FXIIa–CTI complex was analysed with pisa 37, and hydrogen bonding and salt bridge interactions are shown in Table S2.

### Cloning and site‐directed mutagenesis

CTI cDNA was cloned into the pCOLD I‐GST vector 38. The resulting construct encoded a recombinant fusion protein composed of a His_6_ tag, an FXa site, a glutathione‐*S*‐transferase (GST) tag, an HRV3C protease site, and CTI (recombinant CTI [rec‐CTI]). A forward and reverse primer pair for each of the mutations was designed with the www.agilent.com/genomics/qcpd tool, and the mutagenesis was carried out with the Agilent Technologies (Stockport, UK) site‐directed mutagenesis kit. The mutagenesis was confirmed by restriction analysis and DNA sequencing.

### Expression, purification, and characterization

The relevant CTI construct was transfected into expression strain Origami 2 (DE3) and grown under antibiotic selection at 37 °C until an OD of 0.9 was reached. The culture was then induced with 500 μm isopropylthiogalactoside, and incubated for 16 h at 10 °C. Bacterial pellets were collected and lysed by sonication in 100 mm sodium phosphate buffer (pH 7.4), 300 mm NaCl, and 10% glycerol. Purification was performed with Ni^2+^–nitrilotriacetic acid agarose column affinity chromatography. Beads were washed in binding buffer (20 mm sodium phosphate buffer [pH 7.4], 500 mm NaCl, and 20 mm imidazole), and eluted with binding buffer containing 500 mm imidazole. The protein concentration was quantified 39 and verified by equal loading on an SDS‐PAGE gel. Where indicated, protein was purified further by gel filtration chromatography in 50 mm Tris‐Cl and 150 mm NaCl (pH 7.4) on a HiLoad 16/600 Superdex 200 preparative grade column with a fast protein liquid chromatography system (GE Healthcare, Little Chalfont, UK).

Recombinant CTI and CTI(R34A) (0.15 mg mL^−1^ in 50 mm Tris‐Cl, 50 mm NaCl, pH 7.4) were analyzed against buffer by circular dichroism (CD) spectroscopy from 190 nm to 260 nm in a Chirascan‐plus spectrometer (Applied Photophysics, Leatherhead, UK) at 20 °C. Secondary structure was then predicted with circular dichroism analysis using neural networks software 40.

### α‐FXIIa enzymatic assay

The enzymatic activity of α‐FXIIa was measured by monitoring the amount of pNA chromophore released from substrate H‐d‐Pro‐Phe‐Arg‐pNA (S‐2302). Assays were performed in a 100‐μL volume at 33 °C in 96‐well plates in a Perkin Elmer (Seer Green, UK) Envision plate reader, and pNA release was followed over a period of 6 h by reading the absorbance at 405 nm. Absorbance values were converted to pNA concentrations by comparison with a standard curve obtained under exactly the same instrument conditions. All absorbance values were within the linear measurement range of the instrument. Initial rates were calculated on the basis of the first 30 min of incubation. To assess the *K*_M_, concentrations of substrate peptide between 0.1 mm and 0.6 mm were incubated with 10 nm α‐FXIIa, enzymatic activity was measured as described above, and data were analyzed by non‐linear regression with the Michaelis–Menten algorithm (graphpad prism, version 6.04). To assess the inhibitory mechanism of CTI, 30 nm CTI was incubated with these same concentrations of substrate peptide, the reaction was initiated by the addition of 10 nm α‐FXIIa, enzymatic activity was measured as described above, and data were analyzed by non‐linear regression with the Michaelis–Menten algorithm (graphpad prism, version 6.04) and by linear regression after Lineweaver–Burk transformation. To assay inhibitors, concentrations of CTI variant proteins between 10^−8^ m and 10^−5^ m or concentrations of peptides between 10^−6^ m and 10^−3^ m were incubated with 200 μm substrate peptide, the reaction was initiated by addition of 10 nm α‐FXIIa, enzymatic activity was measured as described above, and IC_50_ values were determined by non‐linear regression analysis (graphpad prism, version 6.04) using the log[inhibitor] versus response – variable slope algorithm with a bottom constraint.

## Results

### Docking of CTI and FXIIa

**Figure 1:**
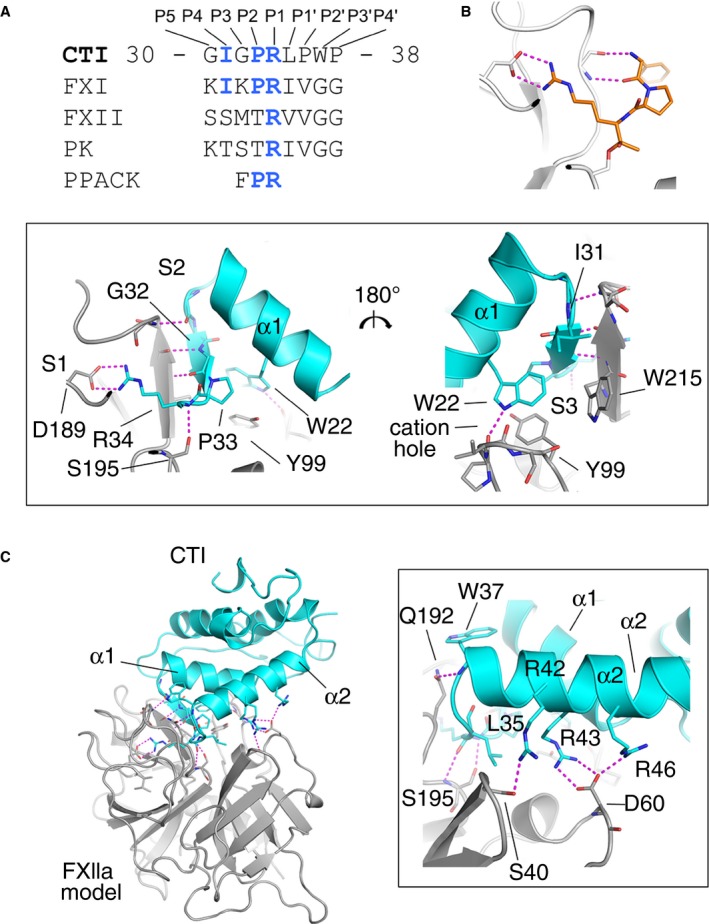
Docking of the activated factor XII (FXIIa)–corn trypsin inhibitor (CTI) complex. (A) The canonical inhibitor sequence of CTI loop residues 30–38 is shown aligned to the sequences of the activation loops of FXI, prekallikrein, and FXII which are natural substrates for FXIIa cleavage, and the sequence of peptidomimetic thrombin inhibitor PPACK. (B) Cartoon diagram showing the canonical inhibitor PPACK binding into the S1 pocket of thrombin prepared by use of the crystal structure with Protein Data Bank code 1PPB with pymol. (C) Cartoon diagram of the docked FXIIa–CTI complex. FXIIa is in gray and CTI is in cyan. Key residues are shown as sticks, and interactions are indicated as purple dotted lines. The top boxed area is a zoom‐in of the area of the FXIIa S1, S2 and S3 pockets (gray) and CTI (cyan), and the right‐hand boxed area is the front of the S1 pocket and the FXIIa H1 pocket residue D60 shown interacting with CTI (residue number D60 corresponds to FXII Asp397 and Asp416 in sequence numbering without and with the signal peptide, respectively, or FXII Asp60A from chymotrypsin numbering).

Previous studies on CTI cleavage by trypsin identified a scissile bond in CTI between Arg34 and Leu35 24 . This, together with sequence similarities between the CTI region of Arg34 and the activation loop of FXI, the natural FXIIa substrate (Fig. 1A) 41, indicates that CTI is likely to be a canonical inhibitor of FXIIa that interacts with the protease in a similar way to the substrate. Kinetic analysis of the inhibition of α‐FXIIa by CTI showed a competitive inhibitory mechanism, in line with this notion (Fig. S3). Kinetic parameters were close to those reported historically for this enzyme (Fig. S3) 14, 42. Figure 1B shows an early crystal structure of a canonical interaction between the peptidic inhibitor PPACK and thrombin 43; however, a non‐canonical model of FXIIa inhibition by CTI has recently been proposed 29. To investigate this, we first produced a homology model of the FXIIa protease, and then performed molecular docking in cluspro, employing this homology model together with the established CTI crystal structures 23, 24.

Inspection of the top‐ranked docking solution identified a canonical complex whereby the CTI residue Arg34 was inserted into the S1 pocket of FXIIa for both CTI crystal structures, with scores of − 999.6 and − 1007.9 and without steric clashes (Fig. 1C shows 1BFA, which is illustrated in stereo in Fig. S2 and Movies S1 and S2). For comparison and to establish the stability of the cluspro docking algorithm, we performed similar docking of CTI with the crystal structures of the protease domains from trypsin, HGFA, thrombin, FXa, FXIa, FIXa, FVIIa, and kallikrein. In each case, the top 10 lowest‐energy solutions were inspected in pymol for canonical complexes in which the Arg34 occupies the S1 binding pocket. The ranked position observed is indicated in the c‐rank column of Table S1. Dockings for trypsin and HGFA with the CTI structure 1BFA gave ranking scores of − 815.6 and − 941.5, respectively, for the canonical complex. As CTI is a known trypsin inhibitor, this result is consistent with previous observations. To our knowledge, inhibition of HGFA by CTI has not been assessed directly. For thrombin and FXIa, a canonical docking pose with CTI was also observed, but only for CTI structure 1BEA, and inspection of these docking solutions revealed steric clashes. This is consistent with previous reports that CTI weakly inhibits FXIa and does not inhibit thrombin 14, 29. Of all the proteases investigated, FXIIa was the only canonical top‐ranked docking solution for both CTI structures 1BFA and 1BEA, and the values of energy were more significant than with any of the other solutions in the study (Table S1).

### Structure of the FXIIa protease–CTI complex

**Figure 2:**
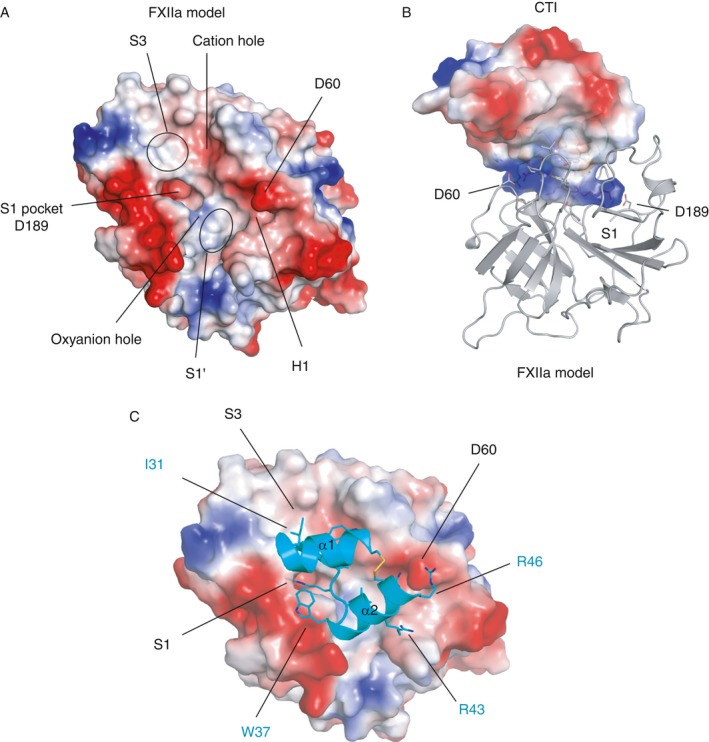
Charge surface representations of the activated factor XII (FXIIa)–corn trypsin inhibitor (CTI) interaction (blue = positive, red = negative). (A) The FXIIa model is shown with the key surface pockets labeled. (B) Docked complex of FXIIa (shown as a cartoon in gray) and CTI shown as a transparent charged surface illustrating the basic region interacting with the negatively charged S1 (D189) and H1 (D60) residues shown as sticks. (C) FXIIa protease as viewed in (A) but with CTI residues 20–44 shown as a cartoon in cyan, with key residues labeled as sticks in cyan.

Figures 1B and 2 show a cartoon diagram of the structure of the FXIIa–CTI docked complex, and Table S2 shows the electrostatic and hydrogen bonding interactions at this interface. The interactions involve salt bridges, hydrogen bonds and hydrophobic interactions burying a total surface area of 1102 Å^2^ (pdbe‐pisa). The Arg34 side chain extends into the S1 pocket of the FXIIa catalytic domain, forming a salt bridge with the S1 pocket residue Asp189 side chain carboxyl (chymotrypsin numbering used) (Figs. 1B and 2; Movie S1). A nitrogen atom from the Arg34 guanidinium group forms a hydrogen bond with the FXIIa Gly218 carbonyl. The main chain carbonyl oxygen of Arg34 forms hydrogen bonds with the main chains of Gly193 and Ser195 in the FXIIa oxyanion hole. In addition, the side chain hydroxyl group of Ser195 from the catalytic triad contacts the Arg34 carbonyl. Additional interactions flanking the P1 Arg34 include Pro33 interacting with the S2 pocket, and the Leu35 side chain forming hydrophobic contacts with the FXIIa S1′ pocket (Fig. 1B; Movie S1). The interactions of CTI residues Gly32 and Pro33 with the FXIIa S2 pocket are similar to interactions observed in other serine protease complex crystal structures with the chloromethylketone inhibitor PPACK 43 (which has the peptidomimetic sequence PheProArg) and protein inhibitors such as infestin‐1 44.

### Loss of α‐FXIIa inhibition for CTI mutant R34A

**Figure 3:**
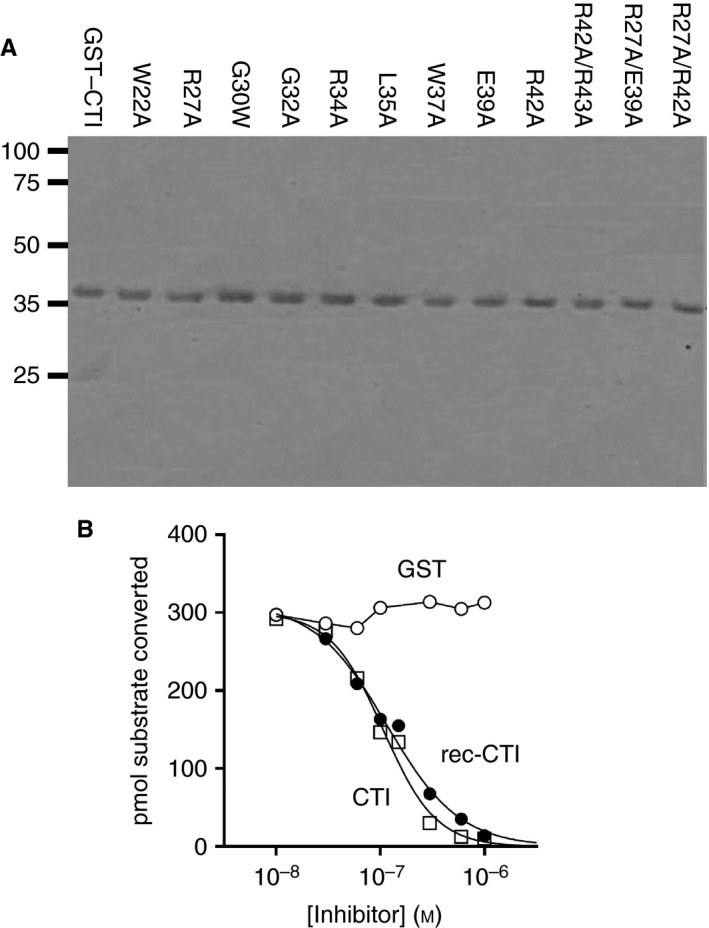
Expression of recombinant corn trypsin inhibitor (CTI) mutants and activated factor XII (α‐FXIIa) activity in the presence of recombinant CTI (rec‐CTI), commercial CTI, and glutathione‐*S*‐transferase (GST). (A) Wild‐type CTI or mutants were expressed as His‐GST fusion proteins, as explained in Materials and methods. Purified proteins were quantified by the Lowry method, and 1 μg of protein was analyzed by SDS‐PAGE followed by Coomassie blue staining. (B) CTI samples (10^−8^ m to 10^−5^ m) were incubated with 200 μm substrate peptide, and this was followed by addition of α‐FXIIa; enzymatic activity was then monitored as described in Materials and methods. Data points were fitted to a curve by non‐linear regression (graphpad prism 6.04; log[inhibitor] versus response – variable slope algorithm with a bottom constraint). Error bars indicate the standard error (*n* = 3–5 independent observations).

**Figure 4:**
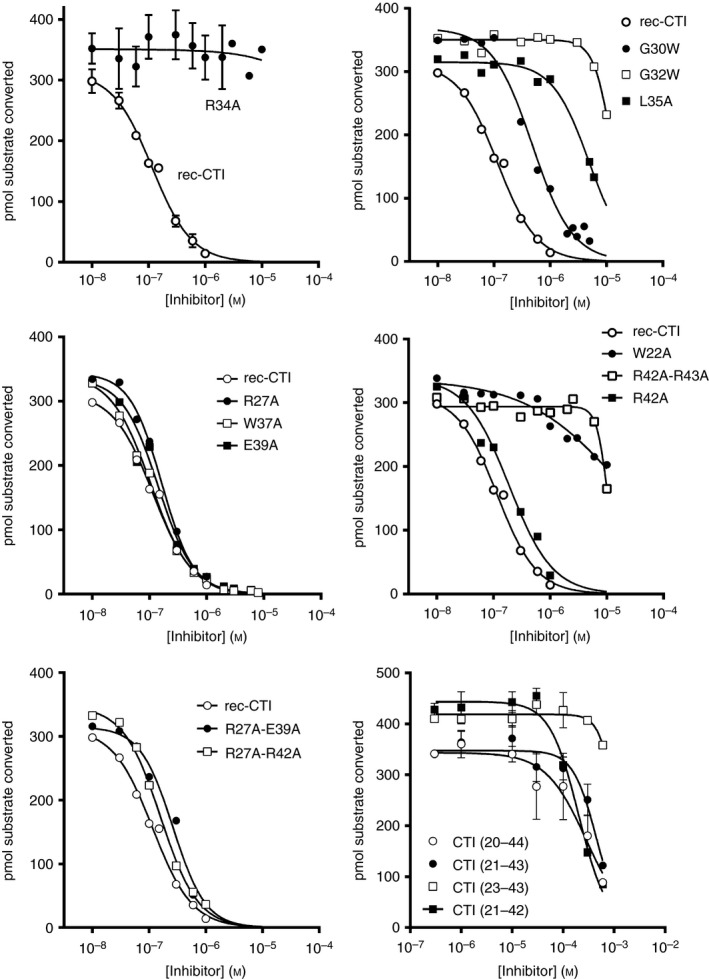
Inhibition of activated factor XII (α‐FXIIa) by recombinant corn trypsin inhibitor (rec‐CTI) variants and peptides. Concentrations of rec‐CTI variants (10^−8^ m to 10^−5^ m) or peptides (10^−6^ m to 10^−3^ m) were incubated with 200 μm substrate peptide, and this was followed by addition of α‐FXIIa; enzymatic activity was then monitored as described in Materials and methods. pIC_50_ values were obtained by non‐linear regression (graphpad prism 6.04; log[inhibitor] versus response – variable slope algorithm with a bottom constraint). Error bars indicate the standard error (*n* = 3–5 independent observations).

**Figure 5:**
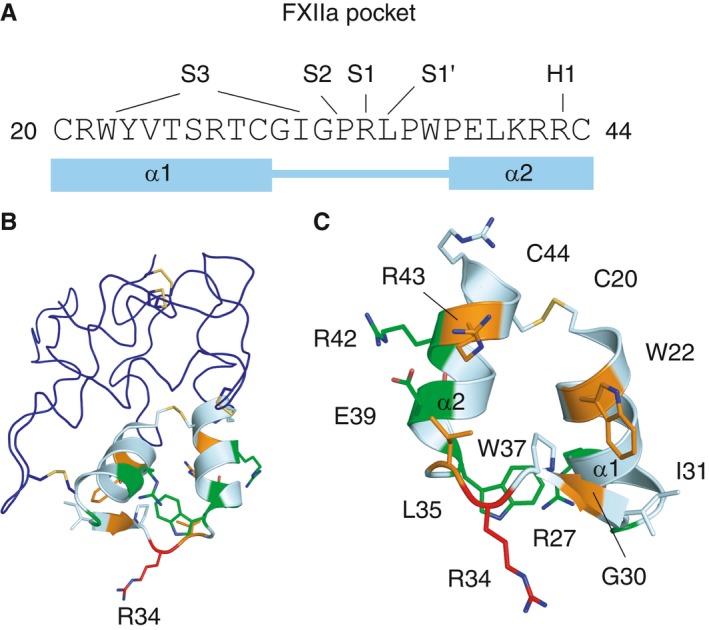
Corn trypsin inhibitor (CTI) substitutions affect inhibition of activated factor XII (FXIIa) to different degrees. (A) The CTI amino acid sequence for residues 20–44 with the respective contact pockets in FXIIa indicated above. (B) Cartoon diagram of the CTI structure showing residues affected by the mutagenesis experiments. (C) Close‐up view of the structure of CTI residues 20–44. Color coding in B and C: Red ‐ essential for binding; Green ‐ important for binding; Orange ‐ contributes to binding.

To test the predictions of the model above, we produced rec‐CTI and utilized mutagenesis to generate 12 variants with amino acid substitutions at the predicted FXIIa–CTI interface (Fig. 3A). rec‐CTI and a commercial preparation of CTI inhibited α‐FXIIa in identical ways, with pIC_50_ values of 6.94 ± 0.04 and 6.95 ± 0.05, respectively (Fig. 3B; Table 1). GST, the main fusion partner of CTI in this construct, did not inhibit α‐FXIIa. Substitution of Arg34 of CTI, at the P1 substrate site, with Ala (R34A) caused complete loss of inhibition of α‐FXIIa, even at high protein concentrations (Figs. 4 and 5; Table 1). CD spectroscopy revealed no differences between wild‐type and rec‐CTI(R34A), with the expected 58% α‐helix secondary structure being present in both (Fig. S4). Gel filtration of wild‐type and rec‐CTI(R34A) revealed that the majority of the proteins appeared as monomers, with a smaller proportion eluting at a size consistent with a dimeric form, most likely because of dimer formation at the GST moiety, with no protein aggregates being visible (Fig. S5). Like the original preparation, the gel‐filtrated monomeric form of rec‐CTI(R34A) was still not able to inhibit α‐FXIIa protease activity, whereas wild‐type rec‐CTI inhibited α‐FXIIa in a similar fashion as the non‐gel‐filtrated protein (Fig. S5). Thus, the lack of inhibitory activity of the R34A mutant was not attributable to loss of protein because of aggregation. This confirms that Arg34 plays a central role in the binding of CTI to FXIIa, and is consistent with the docking showing that Arg34 undergoes the greatest number of interactions with the FXIIa protease (Figs 1B and 2; Table S2) 23, 24.

**Table 1:** Inhibition of activated factor XII by corn trypsin inhibitor (CTI), and recombinant CTI variants. pIC_50_ values are given with the standard error (SE) of the fit (*n* = 3–5 independent experiments). For reference, the corresponding IC_50_ is also given, as is the fold reduction in inhibitory activity of each mutant as compared with inhibition by recombinant CTI (rec‐CTI)

Protein	pIC_50_ ± SE	IC_50_ (m)	Fold
CTI	6.97 ± 0.04	1.1 × 10^−7^	
rec‐CTI	6.94 ± 0.05	1.2 × 10^−7^	1
GST	NIA	NIA	–
W22A	4.72 ± 0.24	1.9 × 10^−5^	158
R27A	6.78 ± 0.04	1.7 × 10^−7^	1
G30W	6.32 ± 0.06	4.8 × 10^−7^	4
G32W	4.89 ± 0.06	1.3 × 10^−5^	108
R34A	NIA	NIA	–
L35A	5.31 ± 0.05	4.9 × 10^−6^	41
W37A	6.97 ± 0.06	1.1 × 10^−7^	1
E39A	6.85 ± 0.06	1.4 × 10^−7^	1
R42A	6.72 ± 0.15	1.9 × 10^−7^	2
R42A/R43A	4.98 ± 0.02	1.1 × 10^−5^	100
R27A/E39A	6.58 ± 0.08	2.6 × 10^−7^	2
R27A/R42A	6.80 ± 0.05	1.6 × 10^−7^	1

GST, glutathione‐*S*‐transferase; NIA, no inhibitory activity was observed.

### CTI substitutions W22A, G32W, L35A and R43A reduce its inhibitory activity

Alanine substitution of Trp22 in rec‐CTI resulted in a 158‐fold reduction in inhibitory activity, showing that it makes a significant contribution to FXIIa binding (Figs 4 and 5; Table 1). This further validates the docking model shown in Fig. 1B, whereby the CTI Trp22 aromatic side chain undergoes a π–π stacking interaction with the FXIIa Tyr99 side chain, and the Trp22 side chain nitrogen atom contacts the Pro96 carbonyl of the cation hole in the S3 pocket. The R42A/R43A double mutant had 100‐fold lower activity than wild‐type CTI. This appears to be mostly attributable to the R43A substitution, as the activity of the R42A mutant was comparable to that of the wild type. This is compatible with our model, which shows that Arg43 forms a salt bridge with FXIIa Asp60A, which would be lost in the R43A mutant (the Asp60A numbering is based on homology with chymotrypsin, with the letter A referring to the first residue of a three‐residue insertion present in FXII but not in chymotrypsin 45; this residue number corresponds to FXII Asp397 without and Asp416 with the signal peptide, and is labeled as D60 in Figs 1 and 2).

Replacement of Gly32 with tryptophan resulted in a 108‐fold reduction in the inhibitory activity of rec‐CTI (Fig. 4; Table 1). Figure 1B shows that the Gly32 main chain nitrogen forms an antiparallel β‐strand hydrogen bond with the FXII Gly216 carbonyl, and this close association is probably disrupted by the introduction of the bulky tryptophan side chain, which may also disrupt contacts made in the S3 pocket by CTI residues Ile31 and Trp22. For comparison, replacement of Gly30 with a tryptophan resulted in only a four‐fold reduction in inhibitory activity (Fig. 4; Table 1). Finally alanine substitution of Leu35 (L35A) resulted in a 41‐fold reduction in the inhibitory activity of rec‐CTI (Fig. 4; Table 1). This is probably attributable to the loss of hydrophobic interactions with the FXIIa protease S1′ pocket, as alanine has an unbranched Cβ.

### CTI substitutions R27A, W37A, E39A, R42A do not affect its activity

Alanine substitution of Arg27, Trp37, Glu39 and Arg42 in rec‐CTI did not affect its inhibitory activity, and the mutants behaved in an identical fashion to the wild type (Figs 4 and 5; Table 1). To further confirm that these residues make a limited contribution to the activity, R27A/E39A and R27A/R42A double substitutions were performed, and these did not influence rec‐CTI activity either (Figs. 4 and 5; Table 1). This indicates that Arg27, Trp37, Glu39 and Arg42 are unlikely to contribute to the interface. For Arg27, this is consistent with the docking pose, as this residue is on the opposite side of the CTI structure to the predicted interface. However, it rules out a more unlikely inverted CTI orientation, which some docking poses suggested could be possible. CTI residues Glu39 and Arg42 form an internal salt bridge in helix α2 and, although the docking predicts a potential hydrogen bond between Arg42 and the FXII Ser40 side chain, the lack of effect of the E39A and R42A substitutions suggests that these do not contribute to CTI inhibition. The Trp37 side chain nitrogen is predicted to form a hydrogen bond with the Gln192 side chain, but replacement of Trp37 with an alanine did not affect enzyme inhibition, indicating that this residue makes little or no contribution to the interface.

### Loss of activity of inhibition loop‐based peptides

The above studies revealed the importance of the 24‐residue stretch Cys20–Cys44 of CTI to the interaction with FXIIa. We employed synthetic peptides from this region to assess whether these had any inhibitory activity against α**‐**FXIIa. A synthetic peptide spanning Cys20–Cys44 and similar peptides lacking N‐terminal or C‐terminal residues were between three and four orders of magnitude less potent than full‐length rec‐CTI (Fig. 4; Table 2). An eight‐mer peptide spanning the loop between helices α1 and α2 (CTI residues 31–38) but containing Arg34 did not have any inhibitory activity (Fig. 4; Table 2). Thus, loss of inhibitory activity was observed when the CTI structure was truncated, with no activity being retained in a core sequence even though critical residues defined by mutagenesis were represented. Weak inhibitory activity of the isolated CTI peptides as compared with the whole protein indicates that the entire CTI protein is required for inhibition. The angle between two α‐helices is likely to play an important role in the interaction with FXIIa.

**Table 2:** Inhibition of activated factor XII (α‐FXIIa) by synthetic peptides based on the central inhibition loop of corn trypsin inhibitor (CTI). Concentrations of inhibitory peptide between 10^−6^ m and 10^−3^ m were incubated with substrate peptide, and this was followed by addition of α‐FXIIa. The initial rate of substrate conversion was determined by following the release of pNA colorimetrically. Data were analyzed by non‐linear regression in graphpad prism 6.04 with the log[inhibitor] versus response – variable slope algorithm with a bottom constraint. pIC_50_ values are indicated with the standard error (SE) of the fit (*n* = 3–5 independent experiments). For reference, the corresponding IC_50_ is also given, as is the fold reduction in inhibitory activity of each mutant as compared with inhibition by recombinant CTI (rec‐CTI)

Peptide	pIC_50_ ± SE	IC_50_ (m)	Fold
rec‐CTI (full‐length reference)	6.94 ± 0.05	1.2 × 10^−7^	1
CTI(20–44): CRWYVTSRTAGIGPRLPWPELKRRC	3.54 ± 0.15	2.9 × 10^−4^	2416
CTI(21–43): RWYVTSRTAGIGPRLPWPELKRR	3.34 ± 0.15	4.6 × 10^−4^	3833
CTI(23–43): YVTSRTAGIGPRLPWPELKRR	2.94 ± 0.05	1.1 × 10^−3^	9166
CTI(21–42): RWYVTSRTAGIGPRLPWPELKR	3.70 ± 0.04	2.0 × 10^−4^	1667
CTI(31–38): IGPRLPWP	NIA	NIA	–
Scrambled: GISTAGPWRRRPPELLKVTY	NIA	NIA	–

NIA, no inhibitory activity was observed.

## Discussion

To investigate the unique protease inhibitory and selectivity properties of CTI, we describe a canonical docked complex of FXIIa and CTI. The model is consistent with the competitive mechanism by which CTI inhibits α‐FXIIa. To test the veracity of the model in more detail, we expressed and purified recombinant variants of CTI, substituting key residues predicted to be important for formation of the interface with FXIIa. These confirm the essential role of the canonical P1 residue Arg34, and establish for the first time the importance of the more remote residues Trp22 and Arg43 in CTI α‐helices 1 and 2. The docking solution is not consistent with a previously reported non‐canonical docking solution 29, which places CTI at a different location on the surface of FXIIa. Differences in the methodology between the two studies are that, in the current study, a hybrid model of the FXIIa structure was used as opposed to a purely HGFA‐derived homology model 29. It is likely that the accurate placement of FXIIa residue Asp60A in the hybrid FXIIa model plays a role in improving the docking as compared with the HGFA‐derived homology model. However, the current canonical model is consistent with experimental observations that disruption of the cysteine loop structure of CTI affects its inhibition of FXII 29, whereby the function of the cysteine loops would primarily be to provide the three‐dimensional context for the conformation of the inhibitory loop of CTI.

**Figure 6:**
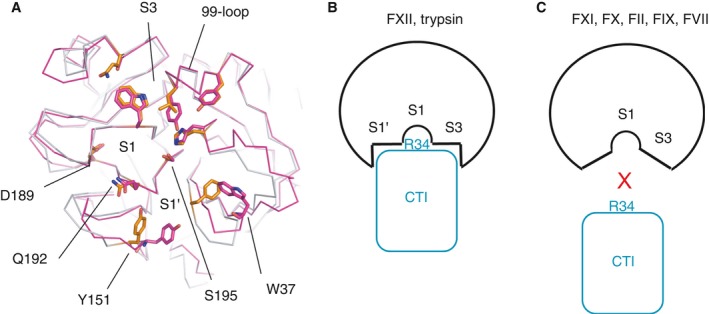
A model for corn trypsin inhibitor (CTI) protease selectivity. (A) Superposition of the activated factor XII (FXIIa) model (purple) and trypsin (gray) shown as a Cα backbone in the region of the S1 and S3 pockets, illustrating the main chain similarity of the 99‐loop. Key side chains are shown as sticks for trypsin (orange) and FXIIa (purple). (B) A combination of features around the S1 and S3 pockets are more open for the bulky CTI inhibitor to bind, with shape changes in the S1 and S3 pockets of other proteases preventing binding of CTI by steric occlusion.

The canonical docking pose that we observed for FXIIa–CTI is similar to the trypsin–CTI and HGFA–CTI complexes predicted by cluspro, albeit that there are fewer interactions overall for trypsin and HGFA, owing to Asp60A (H1 pocket) being absent in these proteases. Superposition of FXIIa and trypsin revealed a striking similarity in the main chain conformation of the 99‐loop between FXIIa and trypsin that is not seen in other proteases (Fig. 6A), albeit that the FXIIa residue Tyr99 is replaced by Leu99 in trypsin. CTI does not inhibit kallikrein, FIXa, and FVIIa 17, and, consistent with this, the docking failed to place CTI Arg34 in the S1 pocket for any of the top 10 solutions examined (Table S1). Although the docking with the FXa protease did produce a solution with CTI Arg34 projecting into the S1 pocket, the Arg34 side chain does not fully enter the pocket and no salt bridge was formed with FXa Asp189, owing to steric clashes around the S1, S2 and S3 pockets. Examination of the CTI–thrombin docked complex revealed that, although Arg34 forms a salt bridge with Asp189, there are steric clashes between CTI and thrombin amino acid side chains in the region of the front of the S1 and S3 pockets; a similar situation was observed for FXIa. One final similarity between FXIIa and trypsin is in the region of the S1′ pocket, where Tyr151 is present in both proteases (as well as in HGFA); it is absent in the other coagulation proteases. The area of the S1′ pocket is hydrophobic in character, with contributions from the side chain of Trp37, and this is utilized by Leu35 of CTI.

Our data indicate that several factors contribute to CTI selectivity for FXIIa over other coagulation proteases, including: (i) the open shape of the S1, S1′ and S3 pockets, accommodating the bulky helical structure of CTI around the P1 Arg34 (Fig. 6B); and (ii) FXIIa side chains Asp60A and Tyr151 in the area of the S1′ pocket, which are missing in other coagulation proteases. A recently described antibody, 3F7, which binds to the FXII protease domain has been shown to be an effective anticoagulation agent 46. This antibody binds via Asp60A, as mutation of this residue to lysine negated 3F7 antibody binding. Another well‐characterized protein inhibitor of FXII that selectively blocks the intrinsic pathway of coagulation is the kissing bug protein infestin‐4, although the molecular basis for its selectivity is unknown. Infestin‐4, expressed as an albumin fusion, provides an effective treatment in murine models of stroke without causing a bleeding side effect 47, 48.

This study used molecular modeling to predict FXIIa–inhibitor interactions. The fact that experimental data can be explained logically by this model underlines its validity. This model could thus be used as a starting point for rational inhibitor design for the treatment of thrombotic cardiovascular disease.

## Addendum

B. K. Hamad, M. Pathak, and R. Manna: contributed to data acquisition, analysis and interpretation of data, preparation of the draft manuscript, and approval of the final version. P. M. Fischer conceived and designed the study, interpreted data, revised the intellectual content, and approved the final version. J. Emsley and L. V. Dekker conceived and designed the study, generated, analyzed and interpreted data, wrote the paper and revised the intellectual content, and generated the final version to be published.

## Disclosure of Conflict of Interests

The authors state that they have no conflict of interest.

## Supporting information


**Fig. S1.** Superposition of the FXIIa hybrid model with the FXIIac and HGFA crystal structures.
**Fig. S2.** Stereo view of a cartoon diagram of the docked FXIIa–CTI complex.

**Fig. S3.** Analysis of the CTI inhibitory mechanism.
**Fig. S4.** CD spectra of wild‐type and mutant (R34A) CTI recombinant protein.
**Fig.S5.** Gel filtration of recombinant CTI and CTI(R34A).

**Table S1.** CTI docking scores and rankings.
**Table S2.** Interfacial hydrogen bonds and salt bridges.

**Movie S1.** FXII–CTI complex and key interactions.

**MovieS2.** Pose of the central inhibition loop of CTI docked into factor XII.
