# Supplementary material for: Assessment of the protein interaction between coagulation factor XII and corn trypsin inhibitor by molecular docking and biochemical validation
Source: J Thromb Haemost. 2017 Aug 9;15(9):1818–28. doi: 10.1111/jth.13773 (PMC5638086; doi:10.1111/jth.13773)
Supplement: Supplementary file 1 — Fig. S1. Superposition of the FXIIa hybrid model with the FXIIac and HGFA crystal structures. Fig. S2. Stereo view of a cartoon diagram of the docked FXIIa–CTI complex. [file JTH-15-1818-s001.pdf]

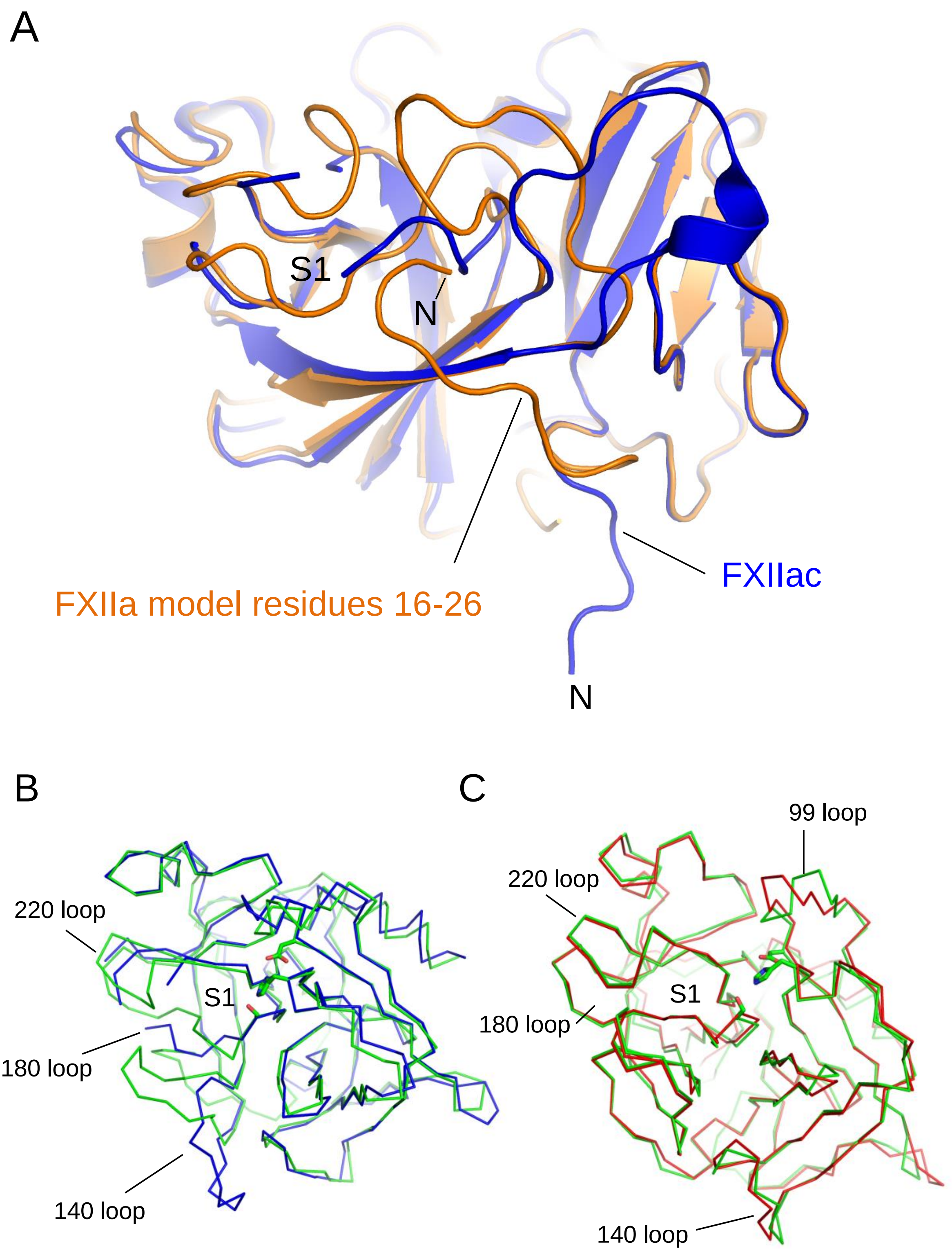

Fig. S1. Superposition of the FXIIa hybrid model with the FXIIac and HGFA crystal structures. (A) Cartoon diagrams showing a superposition of the FXIIa hybrid model (orange) with the FXIIac zymogen like protease crystal structure (pdb:4XE4, blue), with the N-terminal residues indicated. (B) FXIIac zymogen like structure (blue) superposed with the HGFA crystal structure (pdb:1YC0, green) shown as a C-α trace with the key loops labelled and catalytic triad as sticks. (C) The FXIIa hybrid model (red) superposed with the HGFA crystal structure (green) showing differences in the region of the 99-loop.

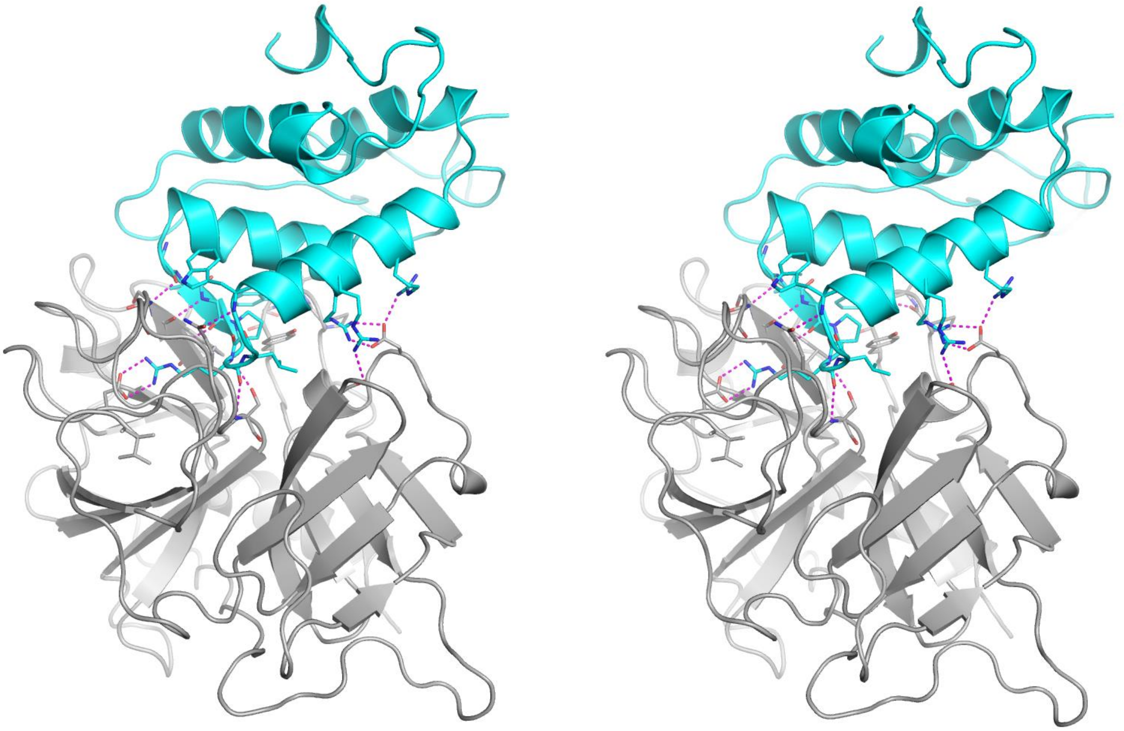

Fig. S2. Stereo view of a cartoon diagram of the docked FXIIa-CTI complex. FXIIa is colored grey and CTI is in cyan. Key residues are shown as sticks and interactions are indicated as purple dotted lines. See also supplementary-movie1 and 2
