# Supplementary material for: Assessment of the protein interaction between coagulation factor XII and corn trypsin inhibitor by molecular docking and biochemical validation
Source: J Thromb Haemost. 2017 Aug 9;15(9):1818–28. doi: 10.1111/jth.13773 (PMC5638086; doi:10.1111/jth.13773)
Supplement: Supplementary file 2 — Fig. S3. Analysis of the CTI inhibitory mechanism. Fig. S4. CD spectra of wild‐type and mutant (R34A) CTI recombinant protein. Fig.S5. Gel filtration of recombinant CTI and CTI(R34A). [file JTH-15-1818-s002.pdf]

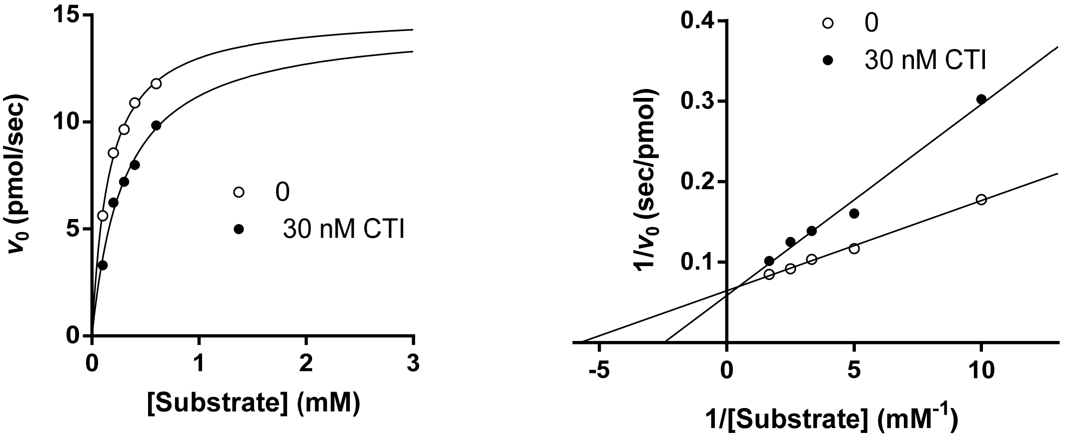

	**0 nM CTI**	**30 nM CTI**
$K_{m\ app}$ (µM)	**162 ± 19**	**301 ± 72**
k_{cat}	**15 ± 1**	**15 ± 2**
K_i (□M)		**29 ± 4**

Figure S3: analysis of CTI inhibitory mechanism

0.1 to 0.6 mM of substrate peptide was incubated without (open symbols) or with 30 nM CTI (closed symbols), after which 10 nM α-FXIIa (equalling to 1 pmol of enzyme) was added and enzyme activity was measured as described in the method section. Data were analysed by non-linear regression (top left panel) and linear regression after Lineweaver Burk transformation (top right panel) and catalytic parameters are summarised in the table..

A

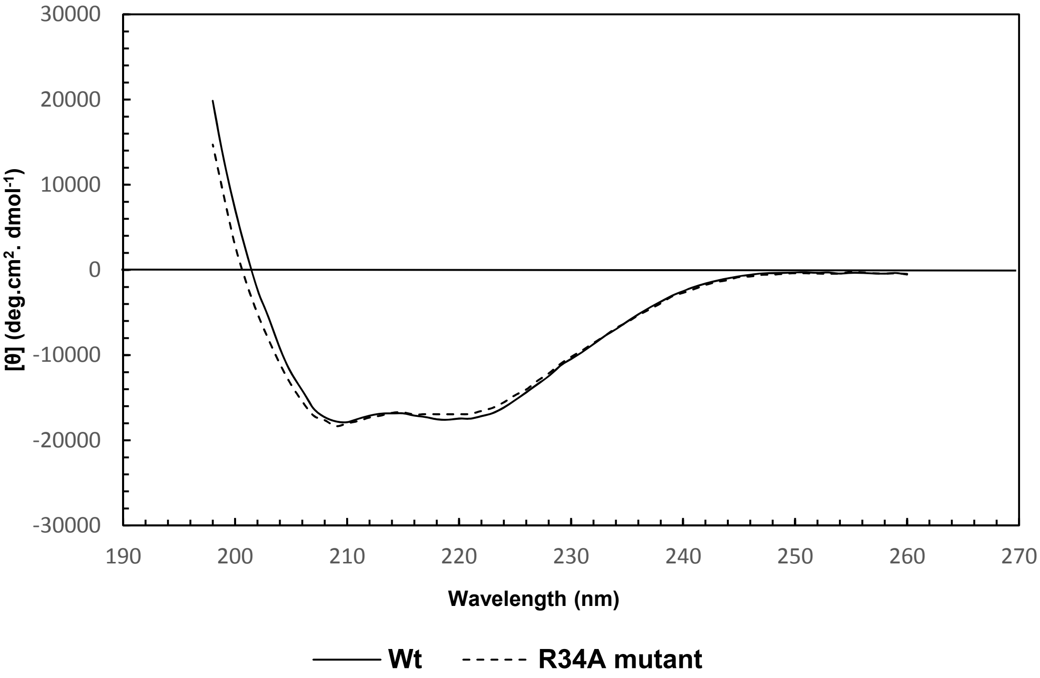

B

	205-260nm	
	**Wt**	**R34A**
Helix	58.4%	58.3%
Antiparallel	4.1%	4.1%
Parallel	4.2%	4.2%
Beta-turn	13.3%	13.3%
Random Coil	19.2%	19.2%
Total Sum	99.1%	99.1%

Figure S4: CD spectra of wild type and mutant (R34A) CTI recombinant protein.

A: Far UV spectrum of recombinant CTI (solid line) and CTI(R34A) (dotted line) samples (0.15 mg/ml). An average of three scans was taken for each sample and a buffer blank sample consisting of CD Buffer alone was subtracted from the averaged spectra. B: Secondary structure estimation from CD spectra predicted using CDNN software. For details see the methods section.

A

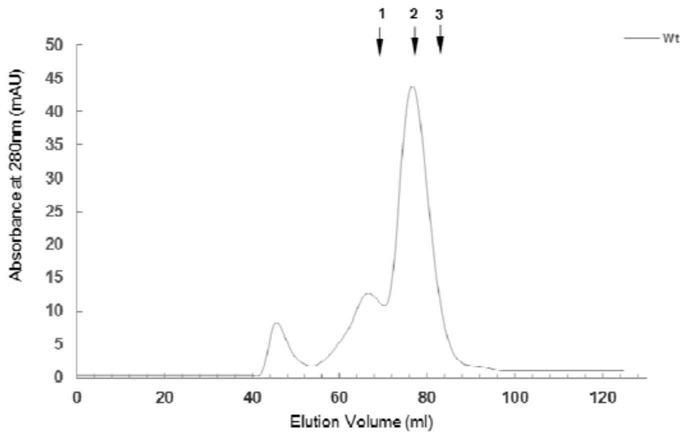

B

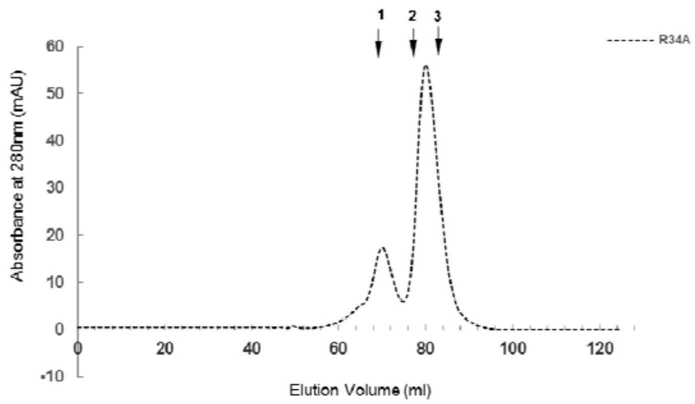

C

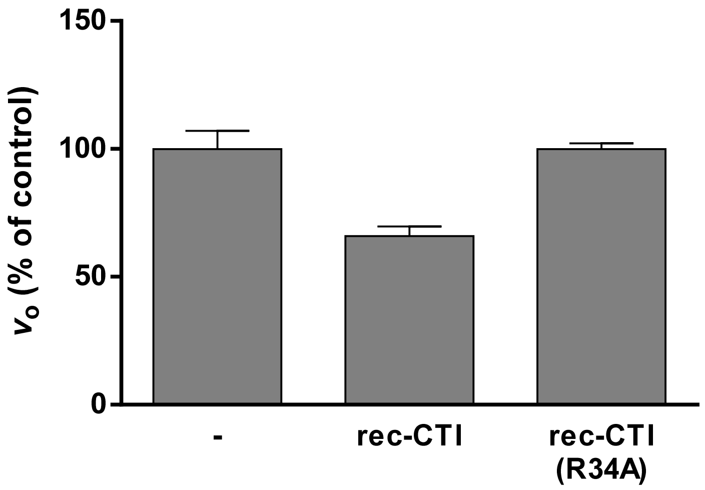

Figure S5: Gel filtration of Recombinant CTI and CTI(R34A)

Recombinant CTI (A) and CTI(R34A) (B) were analysed by gel filtration as described in the methods section. Arrows numbered 1, 2, 3 indicate elution volume of molecular weight standards of 158 kD, 75 kD and 44 kD respectively. (C) 10^{-7} M (final concentration) protein

was incubated with 200 μM substrate peptide followed by addition of 10 nM α-FXIIa.

Velocities are expressed as % of no inhibitor control (n=4).
