# Supplementary material for: Assessment of the protein interaction between coagulation factor XII and corn trypsin inhibitor by molecular docking and biochemical validation
Source: J Thromb Haemost. 2017 Aug 9;15(9):1818–28. doi: 10.1111/jth.13773 (PMC5638086; doi:10.1111/jth.13773)
Supplement: Supplementary file 3 — Table S1. CTI docking scores and rankings. Table S2. Interfacial hydrogen bonds and salt bridges. [file JTH-15-1818-s003.pdf]

**Table S1. CTI docking scores and rankings.**

Two crystal structures for CTI (pdb codes 1BFA and 1BEA) were docked to the target proteases using ClusPro. The top ten docking solutions ranked by ClusPro were considered. The column labelled c-rank denotes the position of the canonical docked complex ranking. For entries marked as unranked, no canonical docking pose was found in the top ten docking solutions. The energy represents the score calculated by ClusPro to rank the docking solutions and values presented in the table are for canonical CTI docking poses only.

| Structure (pdb code) | CTI (1BFA) | | CTI (1BEA) | |
|---|---|---|---|---|
| | c-rank | Lowest Energy | c-rank | Lowest Energy |
| | | | | |
| FXIIa (model) | 1 | -999.6 | 1 | -1007.9 |
| HGFA (1YC0) | 1 | -941.5 | 7 | -946.7 |
| trypsin (4Y0Y) | 1 | -815.6 | 2 | -844.6 |
| thrombin (1PPB) | unranked | - | 4 | -866.2 |
| FXIa (1ZHM) | unranked | - | 9 | -902.6 |
| FXa (2W26) | unranked | - | unranked | - |
| Kallikrein (4ZJ4) | unranked | - | unranked | - |
| FIXa (3LC3) | unranked | - | unranked | - |
| FVIIa (4ZXY) | unranked | - | unranked | - |

**Table S2. Interfacial hydrogen bonds and salt bridges.**

Values were calculated using pdbe-PISA on the FXIIa protease model-CTI docked complex.

| | FXIIa residue[atom] - pocket | Dist. [Å] | CTI residue[atom] |
|---|---|---|---|
| Salt bridges | | | |
| 1 | ASP189[OD2] - S1 | 3.01 | ARG34[NH1] |
| 2 | ASP189[OD1] - S1 | 2.75 | ARG34[NH1] |
| 3 | ASP189[OD2] - S1 | 2.64 | ARG34[NH2] |
| 4 | ASP189[OD1] - S1 | 3.90 | ARG34[NH2] |
| 5 | ASP60A[OD1] - H1 | 3.71 | ARG43[NH1] |
| 6 | ASP60A[OD2] - H1 | 2.74 | ARG43[NH1] |
| 7 | ASP60A[OD1] - H1 | 2.78 | ARG43[NH2] |
| 8 | ASP60A[OD2] - H1 | 2.72 | ARG43[NH2] |
| 9 | ASP60A[OD1] - H1 | 3.91 | ARG46[NE] |
| 10 | ASP60A[OD1] - H1 | 2.88 | ARG46[NH2] |
| Hydrogen bonds | | | |
| 1 | GLY218[H] - S1 | 2.17 | GLY30[O] |
| 2 | GLY216[H] - S2 | 2.02 | GLY32[O] |
| 3 | SER195[OG] - triad | 2.70 | ARG34[O] |
| 4 | GLY193[H] - oahole | 1.76 | ARG34[O] |
| 5 | SER195[H] - oahole | 2.45 | ARG34[O] |
| 6 | GLY216[O] - S1 | 2.02 | GLY32[H] |
| 7 | SER214[O] - S1 | 2.28 | ARG34[H] |
| 8 | GLY218[O] - S1 | 1.68 | ARG34[HH22] |
| 9 | GLN192[OE1] - S1 | 1.94 | TRP37[H] |
| 10 | SER40[OG] - H1 | 1.82 | ARG42[HH21] |
