# Supplementary material for: Assessment of the protein interaction between coagulation factor XII and corn trypsin inhibitor by molecular docking and biochemical validation
Source: J Thromb Haemost. 2017 Aug 9;15(9):1818–28. doi: 10.1111/jth.13773 (PMC5638086; doi:10.1111/jth.13773)
Supplement: Supplementary file 6 [file JTH-15-1818-s006.pdf]

Movie M1: FXII-CTI complex and key interactions

CTI is represented in blue and FXII in grey. M1 is available as a separate movie file, supplementary-movie1, which is playable using Quicktime.

Movie M2: Pose of the central inhibition loop of CTI docked into Factor XII.

FXII is shown in surface representation and the CTI loop as ribbon. M2 is available as a separate movie file, supplementary-movie2, which is playable using Quicktime.
